# Two-Dimensional Eye Movement Characteristics of Infantile Nystagmus in Eccentric Gaze

**DOI:** 10.1167/iovs.67.12.5

**Published:** 2026-10-02

**Authors:** Onyekachukwu M. Amiebenomo, Matt J. Dunn, Lee McIlreavy, Jonathan T. Erichsen, J. Margaret Woodhouse

**Affiliations:** 1 School of Health and Social Wellbeing, University of the West of England, Bristol, United Kingdom; 2 School of Optometry and Vision Sciences, Cardiff University, Cardiff, United Kingdom

**Keywords:** fixation, visual acuity, null zone, vertical eye movements

## Abstract

**Purpose:**

Infantile nystagmus (IN) eye oscillations are usually predominantly horizontal. However, the contribution of the vertical component and its relationship with the horizontal component as a function of gaze angle remain unknown. This study aimed to quantify fixation performance at horizontal gaze angles, in two dimensions.

**Methods:**

Eye-tracking data from 17 participants with IN, fixating nine horizontal gaze positions spanning ±20°, were evaluated using a bivariate probability density function method, wherein the derived 68% isocontour of eye position samples were analyzed to quantify the accuracy and precision of fixation. In addition, the horizontal and vertical axis range (component) of this isocontour was evaluated using linear mixed models to determine how these vary across gaze angles.

**Results:**

From individual plots, the accuracy and precision of fixation varied between participants. There was a significant quadratic effect of gaze angle on the horizontal and vertical axis range of the 68% isocontour (β = 0.005, *P* < 0.001). Across participants, the gaze angle of highest fixation precision and least eye movement intensity showed a small mean difference of 0.29° but high variability. Also, mean values for fixation performance across eccentric gaze angles showed significant positive correlation with clinically measured visual acuity (accuracy: *r* = 0.659, *P* = 0.004; precision: *r* = 0.677, *P* = 0.003).

**Conclusions:**

Using this approach should enhance our understanding of the complexities of the null zone. These findings highlight the individual nature of IN oscillations and suggests that fixation precision may play a role in determining the preferred gaze position. Evaluating two-dimensional eye movements during eccentric gaze provides a more complete understanding of the relationship between fixation performance and visual acuity.

Infantile nystagmus (IN) is a pathologic involuntary movement of the eyes, usually developing within 6 months after birth or (rarely) may be present at birth.^1^ In most cases, IN eye movements are conjugate, symmetrical, and primarily horizontal.^2^^,^^3^ IN is estimated to occur in 1.4 per 1000 of the population,^4^ significantly affecting functional vision.^5^^,^^6^ The null zone in IN is the range of gaze angles within which the IN oscillation is minimized. From the individual's perspective, anecdotal evidence suggests that the null zone is a preferred eye position that the person chooses to adopt, especially when concentrating on a visual task.^7^ When the null zone is at an eccentric gaze position, an abnormal head position (AHP) may develop, presumably to reduce the oscillation and enhance vision. Null zones are found in more than 60% of people with IN,^3^^,^^8^ often at primary position or within ±10° of primary position.^3^

The null zone is an important characteristic of IN—outcome measures of IN treatment often rely on finding and shifting this position centrally through surgical or optical means,^9^^–^^13^ or by maximally utilizing it through a procedure known as null zone training.^14^^,^^15^ This is because, at the null zone, visual acuity (VA) has been shown to be slightly better and retinal slip minimized,^16^^–^^18^ with one study finding a marginal but significant improvement of 0.08 logMAR at the null zone.^16^ In addition, one or more of the other waveform parameters often used to determine fixation performance, such as the amplitude and frequency, as well as the foveation duration and accuracy, are known to significantly improve at the null zone. For example, amplitude and frequency are reduced, fixation duration is lengthened, and accuracy is improved.^16^^,^^18^^,^^19^

There is no universally accepted approach to defining or measuring the IN null zone. Traditionally, the null zone is determined by quantifying one or more of the features of the IN waveform as recorded by eye tracking—specifically, the amplitude, frequency, and/or intensity, as well as slow-phase velocities and foveation duration.^3^^,^^18^^,^^20^^–^^26^ Any one of these methods for evaluating IN eye movements, however, may not give a comprehensive picture of the waveform and may be cumbersome when each IN waveform feature is to be considered and presented individually. For example, amplitude provides a measure of the total excursion of the eye, but does not account for the uneven distribution of eye positions sampled in a cycle, because eye positions during the slow phase, and especially during foveation, are weighted toward the target. Moreover, frequency does not necessarily correlate with the amplitude of the waveform oscillation; that is, waveforms may have similar frequencies but different amplitudes (and vice versa), when the eye position trace is considered.^16^ In other words, a measure of amplitude alone, frequency alone, or the product of both (intensity) quantifies the IN waveform metrics, but might not give the complete picture of the IN waveform oscillation.

Another inherent drawback with traditional IN eye movement analysis methods is the fact that, most of the time, only the horizontal component of the eye movement trace is considered.^2^^,^^16^^,^^20^^,^^22^^,^^27^^–^^34^ This may partly be because the IN oscillation is predominantly horizontal or historically available instrumentation permitted only one axis to be measured at a time.^24^^,^^26^^,^^28^ Although a few studies present both horizontal and vertical components separately,^24^^–^^26^ these approaches still miss out information on how these components relate to each other within an individual. Most modern video-based eye-tracking technology typically allows recording of both the horizontal and vertical axes (but excludes torsional eye movements) and therefore provides the opportunity for simultaneous analysis of these eye movement components.

Traditional waveform metrics of amplitude, frequency, and intensity focus on temporal waveform properties, whereas the bivariate probability density function (bPDF) analysis^35^ quantifies the two-dimensional spatial distribution of eye positions during attempted fixation, irrespective of the waveform. For this study, IN oscillations were quantified during attempted fixation using the bPDF analysis, providing novel information regarding how this relates to gaze angle and VA. The bPDF evaluates relative eye position to produce measures of accuracy and precision of fixation performance from the derived non-uniform isocontour (i.e., the highest 68% density, analogous to ±1 SD of a normal distribution). The bPDF analysis considers the two-dimensional data with the highest probability density while encompassing a portion of the data and excluding outliers; hence, it differs from traditional foveation metrics,^31^^,^^36^ which, again, consider only horizontal eye movements.

In one previous study,^26^ the horizontal and vertical variability of foveations in people with IN were independently evaluated, using data from video recording of participants fixating in a fundus camera. That study reported a significant positive relationship between the horizontal and vertical variability of eye positions (during foveations), as well as a relationship between the horizontal variability and VA (measured using minimum angle of resolution). However, other measured variables (namely, amplitude, frequency, and intensity) did not correlate significantly with VA. This finding highlights the importance of increased precision during foveation to improve vision; therefore, the present study aimed to provide a more comprehensive two-dimensional evaluation of fixational eye movement performance (accuracy and precision) at different angles of gaze. We quantified IN oscillation during attempted fixation and explored the potential use of measures of their accuracy and precision in order to provide a better understanding of the basis for null zone preference. Another aim is to determine the relationship between these measures of fixation and VA.

The dynamics of nystagmus eye movements (e.g., intensity) are known to vary with gaze angle.^16^ People often choose to adopt a gaze angle where these values are at a minimum (i.e., the null zone),^2^^,^^37^^,^^38^ but it remains unclear how that relates to the full function involving two-dimensional eye movements. Therefore, we also wished to investigate if fixation accuracy and precision also change with gaze angle and how the fixation precision measure compares with nystagmus waveform parameters (i.e., amplitude, frequency, and intensity). In the present study, these parameters were used to identify the conventionally defined null zone gaze position for each participant. We then examined whether this position corresponded to the gaze position with the smallest bPDF isocontour area, representing the smallest two-dimensional distribution of eye positions during attempted fixation. This comparison was intended to assess whether the conventionally defined null zone also coincides with the gaze position of greatest two-dimensional fixation precision, rather than to suggest that waveform-derived measures and bPDF precision quantify the same aspect of the eye movement. In line with the previous study^26^ that examined the variability of eye positions and VA, another major interest was whether participants with poor VA exhibit worse eye movement precision overall (i.e., increased bPDF isocontour area values).

## Methods

### Participants

Eye movement data from 17 adults with IN, recruited from an existing volunteer participant cohort of the Research Unit for Nystagmus (RUN) at Cardiff University, were analyzed for this study. Ethical approval was obtained from the School of Optometry and Vision Sciences Research Ethics Audit Committee (project no. 1380). All participants gave informed consent in writing before the study commenced. Inclusion for this study required a diagnosis of IN as reported by the participant or ophthalmologist. This diagnosis was confirmed by an optometrist (MJD) after a detailed history, slit-lamp examination, optical coherence tomography, and high-speed eye movement recording. Participants who were found to have acquired nystagmus or fusion maldevelopment nystagmus syndrome were excluded from the study.

### Procedure

Participants were seated in a darkened room, 1 meter from a large display screen (1.43 × 1.08 m with a 1400 × 1050-pixel resolution; luminance range, 1.5–180 cd/m^2^). Stimuli were rear projected onto the screen by a cathode ray tube (CRT) projector (DS+26; Christie Digital Systems, Cypress, CA, USA). A video-based eye tracker (EyeLink 1000, firmware version 4.594; SR Research, Ottawa, ON, Canada) was set in desktop configuration to record horizontal and vertical gaze coordinate data at 1000 Hz, with the chin and head supported by a SR Research rest. Figure 1 illustrates the setup for this experiment. The eye tracker was calibrated using the method described by Dunn et al.^39^ based on isolation of the foveation durations at the 45 gaze positions recorded during the experiment, each of which were recorded twice (see below).

**Figure 1.**
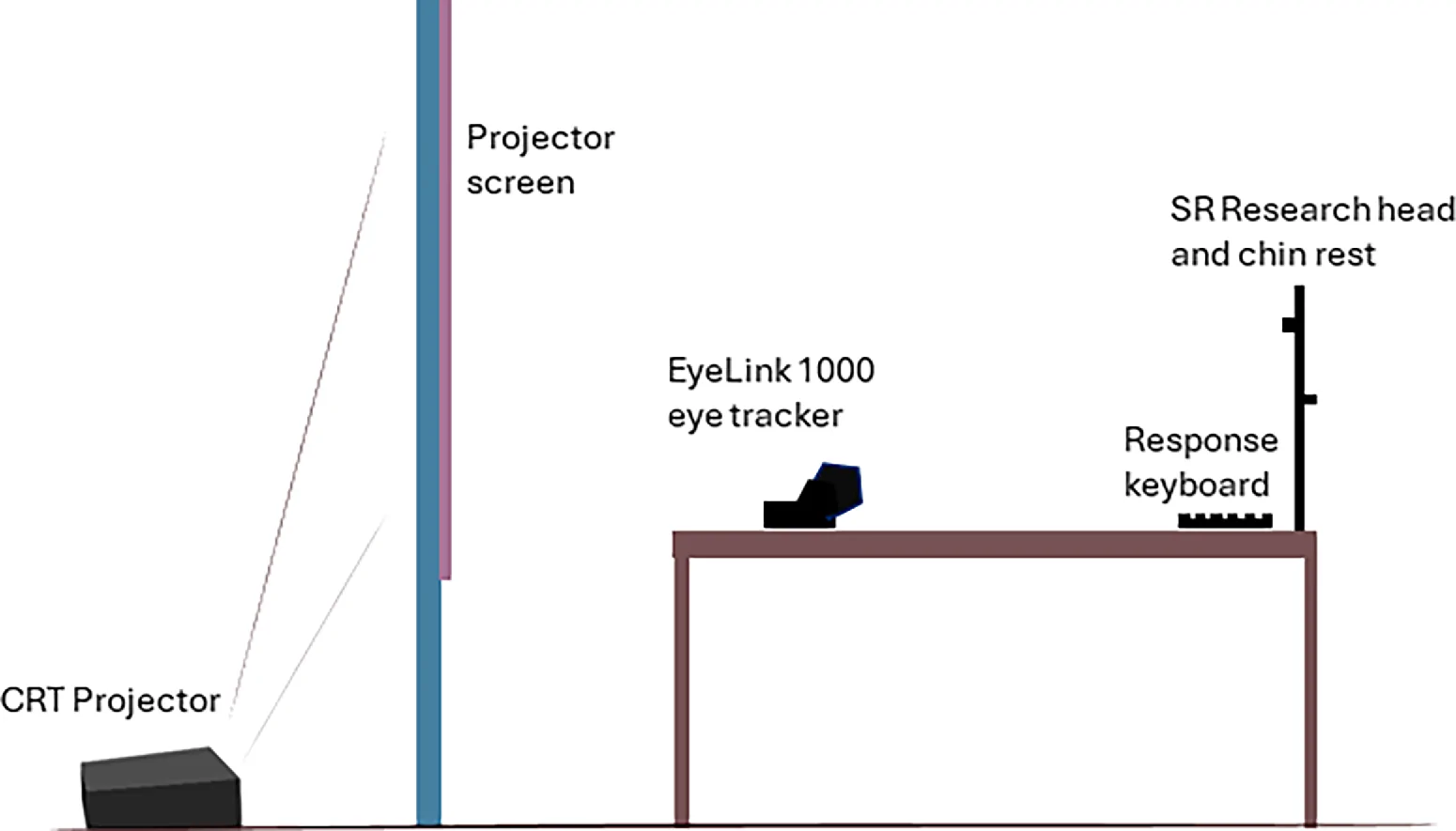
Laboratory set-up.

Participants were comfortably stabilized in the SR Research chin and forehead rest, after which measurements were obtained while fixating a 1° red target^40^ on a black background, shown at 45 gaze positions in a grid spanning ±20° horizontally and vertically (nine horizontal steps at 5° intervals and five vertical steps at 10° intervals). Stimuli were generated in MATLAB R2019b (MathWorks, Natick, MA, USA) using the Psychophysics Toolbox extensions.^41^^–^^43^

Prior to recording eye position, clinical VA was measured using a rear-illuminated 4-meter Early Treatment Diabetic Retinopathy Study (ETDRS) chart (Good-Lite, Elgin, IL, USA). Measurement was performed at 4 meters with fixation in primary position. During measurement, participants were given as much time as they needed to identify the letters. They were encouraged to keep reading until the end point, where at least four letters were missed on a line.

During recording, each position was tested twice; the order of positions was pseudorandomized to compensate for any order effect. With participants wearing any habitual distance refractive correction, eye movements were recorded monocularly from each eye (the fellow eye was patched). The results reported in this paper are limited to data from the nine horizontal positions across the vertical midline, from the eye with the better VA. Where both eyes had equal VA, data from the right eye were analyzed.

### Eye Movement Data Processing

Data files were visually inspected to ensure successful eye movement recording using EyeLink Data Viewer 3.2.1 (SR Research). Blinks (i.e., periods when the pupil can no longer be detected), as well as 100 ms of data before and after blinks,^44^ were first identified in Data Viewer to be excluded at the MATLAB interface. This was necessary to eliminate blink-related artifacts or blink saccades.^44^^,^^45^ In addition, the eye movement traces were visually inspected to ensure there were no saccades included, other than the nystagmus fast phases. Thereafter, saved data were analyzed using MATLAB. Pixel-based gaze data were converted to degrees, where (0,0) denotes screen center and positive values indicate rightward or upward positions.

Each trial for a given stimulus position consisted of 5031 ms of data. Of these, the initial 800 ms of eye position data (value determined empirically) from each stimulus position were removed to allow participants sufficient time to redirect their gaze toward the new target. Because data were obtained twice from each position, both sets of data were concatenated. Therefore, after removing the initial 800 ms of data in each case, as well as the blink periods, a total of about 8400 ms of data remained for analysis at each stimulus position. To remove high-frequency noise, eye position (in degrees) was then smoothed using a low-pass fourth-order Butterworth filter with a cut-off frequency of 60 Hz.^46^^,^^47^

### bPDF Analysis

The method used to analyze fixational eye stability and quantify fixation performance in the present study incorporates a bPDF derived from eye position data. The resulting 68% isocontour allowed a further evaluation of the horizontal and vertical components of the eye movement position at a specified gaze direction, irrespective of the type of waveform present, and includes the calculated metrics for the direction of inaccuracy, a specified area of eye positions, and the orientation of imprecision (indirectly accounting for the plane of IN oscillation). The bPDF method for analyzing eye movement traces has been used in recent studies for both typically sighted individuals^48^^,^^49^ and those with IN.^50^

A bPDF analysis involves combining the horizontal and vertical components of eye position data. Eye position was determined relative to the target and computed in two dimensions to produce a non-uniform isocontour,^35^ using an open-source script.^51^^,^^52^ Similar to previous studies,^49^^,^^50^^,^^53^ the preferred isocontour proportion was chosen as the highest 68% density of the entire set of eye position points recorded. This proportion is analogous to taking the mean ± 1 SD and excludes extreme fixational eye movement values (i.e., outliers).

As shown in Figure 2, accuracy was computed as the vector distance of the centroid of the non-uniform isocontour to the target, wherein the smaller this vector distance or closer the centroid to the target, the more accurate the fixation. Therefore, more accurate eye movements are quantified by smaller values for accuracy. Precision, on the other hand, was determined as the variability of recorded eye position data points (i.e., the contour area), as well as the shape factor of the isocontour. A larger isocontour area would indicate that relative eye positions are more spread and hence less precise. The shape factor, which provides more information on the isocontour symmetry, was obtained from the ratio of the minor to major axes, whereby the minor axis indicated the position of least spread in eye positions and the major axis the position of greatest spread in eye position, signifying more imprecision. A value of 1 indicates equal distribution of eye position along both axes (i.e., a circular isocontour).

**Figure 2.**
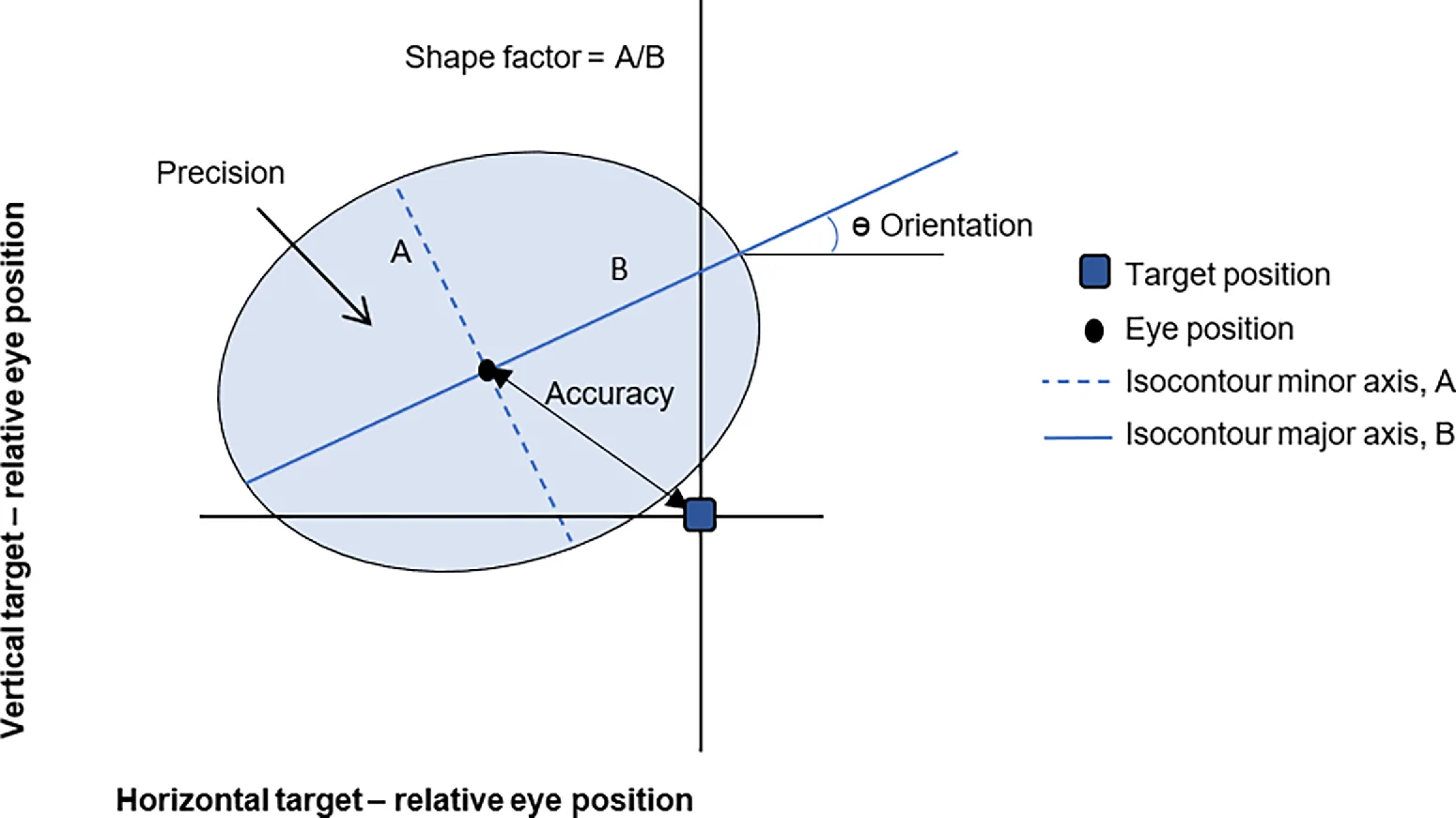
Schematic representation of the metrics used to quantify fixation. The *blue oval* represents the contour with 68% of highest density data points. The major axis (*solid blue line*) represents the region of greatest imprecision, and the angle this axis makes with the horizontal plane provides a measure of orientation.

Values representing the horizontal and vertical axis range (component) of the 68% isocontour were further computed using MATLAB. The major and minor isocontour axes always lie perpendicular to each other. Therefore, the orientation of the isocontour major axis (i.e., the angle between the major axis and the horizontal plane), together with the precision values of contour area and contour shape, were used to derive the horizontal and vertical axis range of the 68% isocontour. Quantitative measures of amplitude, frequency, and intensity were calculated, using custom code in MATLAB, for each gaze angle using values obtained from the two recordings at each measured position.

### Statistical Analysis

A linear mixed model using restricted maximum likelihood was used to assess the effects of gaze angle, fixation axis and their interactions on axis range values. A quadratic gaze angle term (*angle_sq* = *angle*^2^) was included to model the anticipated U-shaped relationship between gaze angle and fixation stability. Participant (*id*) was included as a random effect, with random intercepts and slopes for gaze angle (*angle*). R 4.5.1 (R Foundation for Statistical Computing, Vienna, Austria) and the lme4 package were used to compute the model equation as follows:

$$\begin{array}{ccc} & & axis\_range∼angle+angle\_sq+axis\\ & & \;+\;angle:axis+angle\_sq:axis+\left(1+angle\left|id\right.\right) \end{array}$$

Model singularity and collinearity diagnostics were performed to ensure correctness of model estimates. Statistical significance was defined at *P* < 0.05 for all analyses.

## Results

### Participant Information

Clinical data for all participants are presented in Table 1. Eleven female and six male participants were seen, ages 18 to 72 years (mean, 44.2 ± 16.6 years).

**Table 1.** Participant Clinical Data

Participant	Age (Y)/Sex	Diagnosis	VA (logMAR)
P010	53/F	Idiopathic	0.48
P012	65/M	Achromatopsia	0.98
P016	30/M	Idiopathic	0.50
P018	47/M	Unknown macular defect	0.32
P019	72/M	Idiopathic	0.30
P020	23/F	Idiopathic	0.02
P021	49/F	Albinism	1.36
P022	28/F	Albinism	0.32
P023	25/F	Albinism	1.10
P025	43/F	Idiopathic	0.60
P026	61/M	Albinism	0.52
P027	45/F	Optic atrophy	−0.06
P028	57/F	Idiopathic	0.56
P030	54/F	Optic atrophy	0.80
P031	24/F	Idiopathic	0.32
P033	18/F	Aniridia	1.30
P034	57/M	Idiopathic	0.02

### Fixation Performance at Gaze Positions

Measures of accuracy and precision were used to quantify fixation performance at each gaze position. An example of a participant's eye position and bPDF analysis at three gaze positions is shown in Figure 3. In this participant, the horizontal component of the eye movement trace changes beat direction from one extreme gaze to the other, becoming less consistent at the central fixation position. In addition, the vertical component of the eye movement trace indicates more oscillations at the extreme gaze positions. Furthermore, it can be noted that a change in beat direction may be reflected by a left or right shift in the overall distribution of points in the bPDF plots. In addition, this change in beat direction will cause a change in the isocontour and would be reflected in a much larger area or lower precision value.

**Figure 3.**
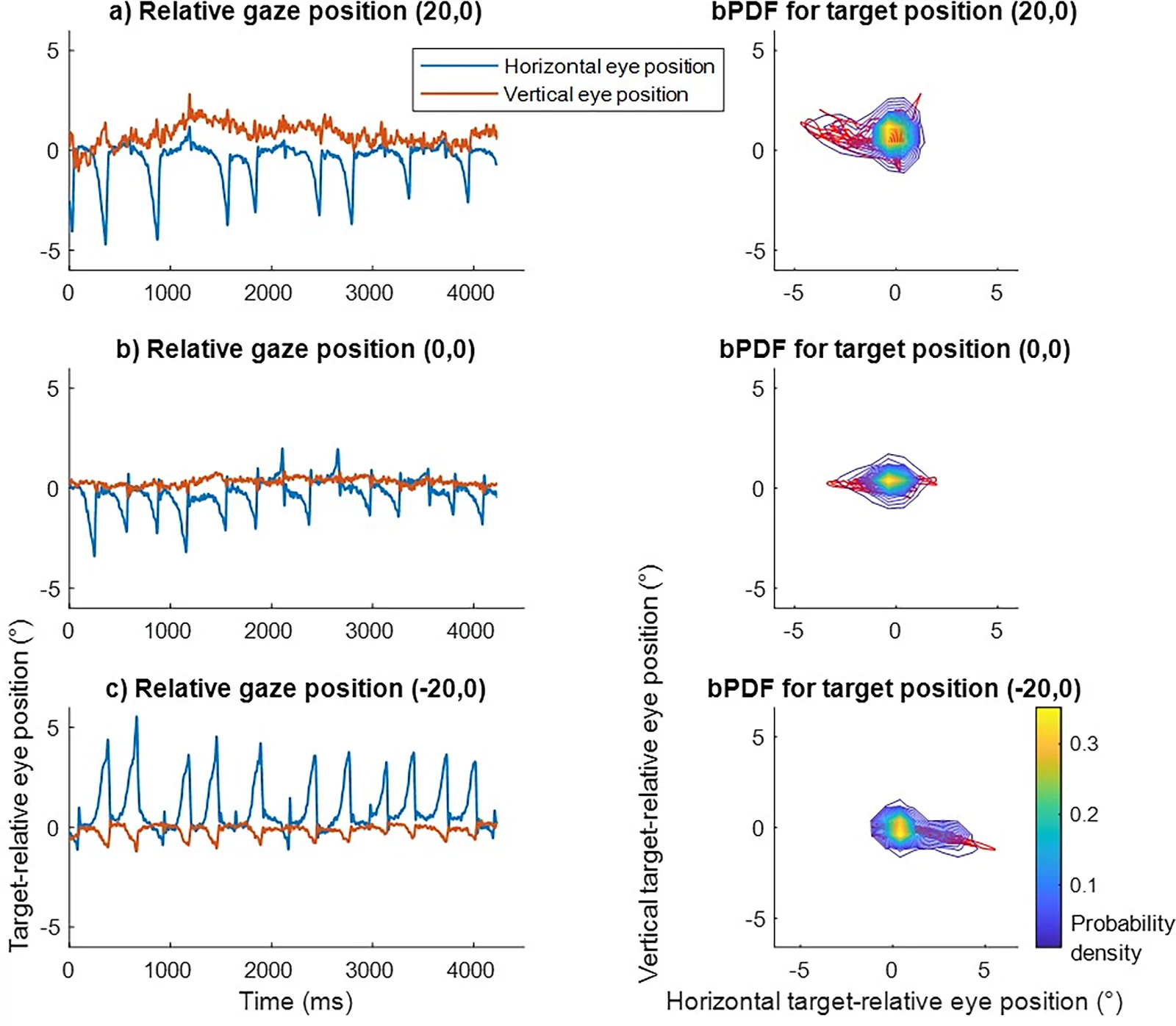
Representative eye position data and corresponding bPDF analysis output for participant P028 while fixating horizontally at +20° (**a**), central (**b**), and −20° gaze angles (**c**). Shown are the target situated 20° rightward with respect to head position (**a**), straight ahead (**b**), and toward the left (**c**). A deflection toward the positive position on the vertical axis indicates a rightward eye movement. Diagrams show a significant Q3 vertical component of IN waveform which neither intensity nor amplitude can capture in a traditional waveform analysis, unless performed for horizontal or vertical eye movement traces differently. The *red lines* underneath the isocontour images on the right-hand side show all recorded eye positions. It is the isocontour surrounding the gaze positions with the highest 68% density that was further analyzed to give measures of fixational eye stability.

It is interesting to note that, in the eye position plots, there was an evident vertical component (especially when viewing at the −20° position). If evaluations of only horizontal eye movement amplitude or intensity were performed, this vertical eye position information would not be captured. The results illustrate the variability of the IN waveform, including the vertical component, at different gaze positions.

The accuracy, contour area, and shape factor (contour shape) derived from the isocontour, at each gaze angle, are plotted for all participants in Figure 4. As described in Methods, smaller values indicate greater accuracy. Larger contour areas indicate a larger spread of gaze positions and hence a greater imprecision; or, in other words, they could reflect a larger “relative” waveform amplitude. The imprecision could be equally spread along the major and minor axes, thereby producing a value close to 1 for the shape factor (ratio of minor to major axis) metrics. A contour shape of 1 indicates that data spread along the major and minor isocontour axes are nearly symmetrical and that the IN waveform oscillation in both components have remained nearly equivalent in magnitude (considering 68% of the highest density points). Where there is a dissimilarity in the data spread in both waveform components, as shown by these participants, this may reflect a change in the spatial distribution of IN eye positions within each participant across gaze angles.

**Figure 4.**
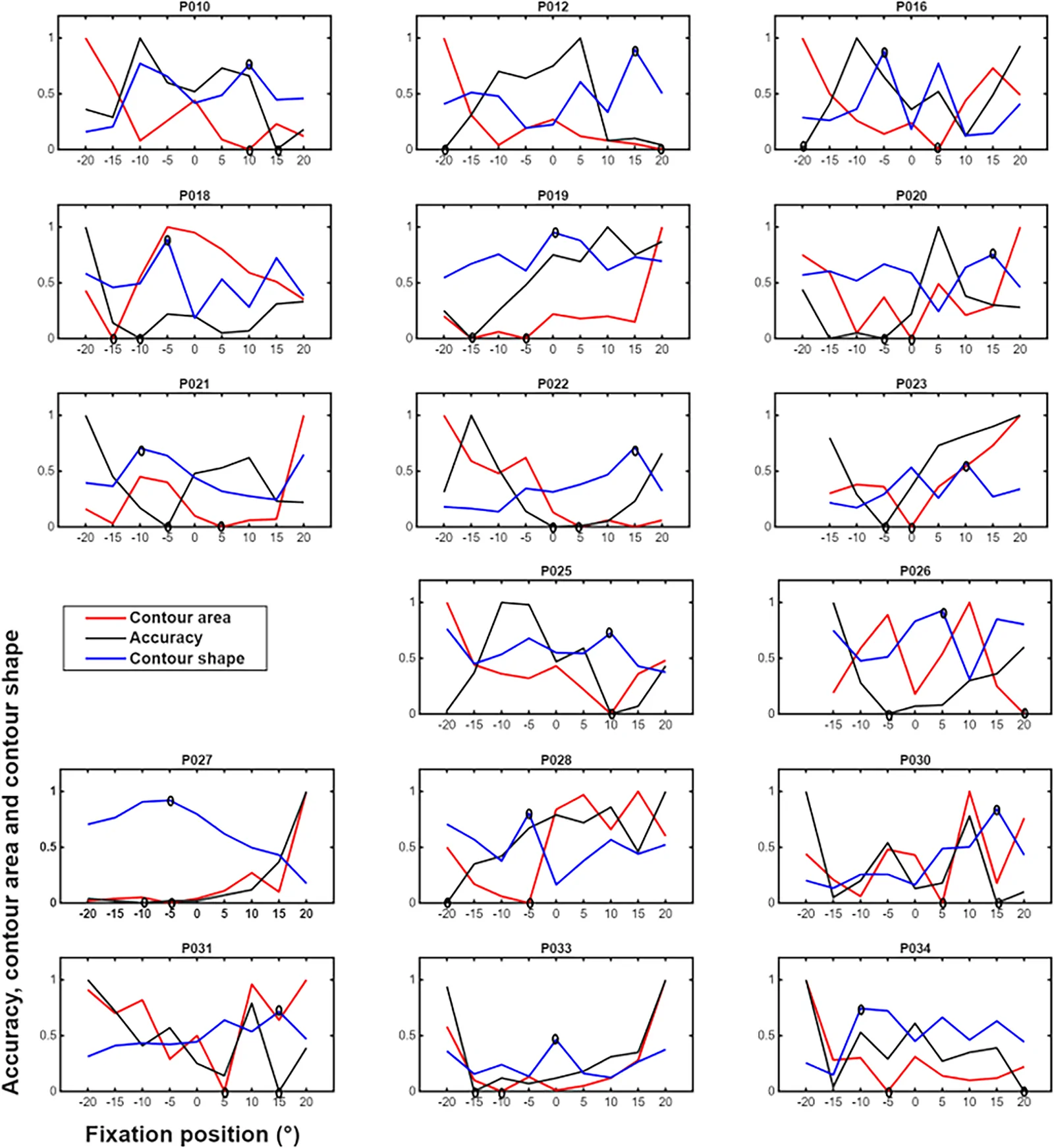
Accuracy, contour area, and contour shape at all fixation positions. Normalized values were plotted for accuracy and contour area. Contour shape values were computed on a scale of 0 to 1 and hence were not normalized. The small *black ovals* in each plot indicate points of highest accuracy and precision including the point of eccentric gaze where the contour shape was closest to 1.

Normalized values for each metric, except contour shape (given that values range already between 0 and 1), are presented in Figure 4 and Figure 5. A summary of the mean real values for each participant is also presented in Table 2. Most participants did not show an obvious systematic change in accuracy or contour area with gaze position, but there were two participants for whom there was a dramatic increase in contour area at extreme gaze: P012 and P021. The real values obtained from these participants changed more than in others and varied from 5.0 to 51.8 and 12.3 to 62.6 (deg^2^), respectively, with the highest values recorded at extreme gaze. These high values warranted normalization of the real data. Normalization was performed per participant (i.e., across nine values obtained at −20 to +20° gaze angles) for contour area and accuracy. Normalization was performed using the following formulae: *x*/range = normalized data, where *x* = data point – minimum data, and range = maximum data – minimum data. Given that the horizontal and vertical axis range values were derived from the contour area, these were normalized as one group of 17 gaze positions rather than in two separate groups. Normalization did not influence the identification of the most accurate and precise gaze positions, as normalization set the lowest data point to zero, making these positions easier to identify.

**Figure 5.**
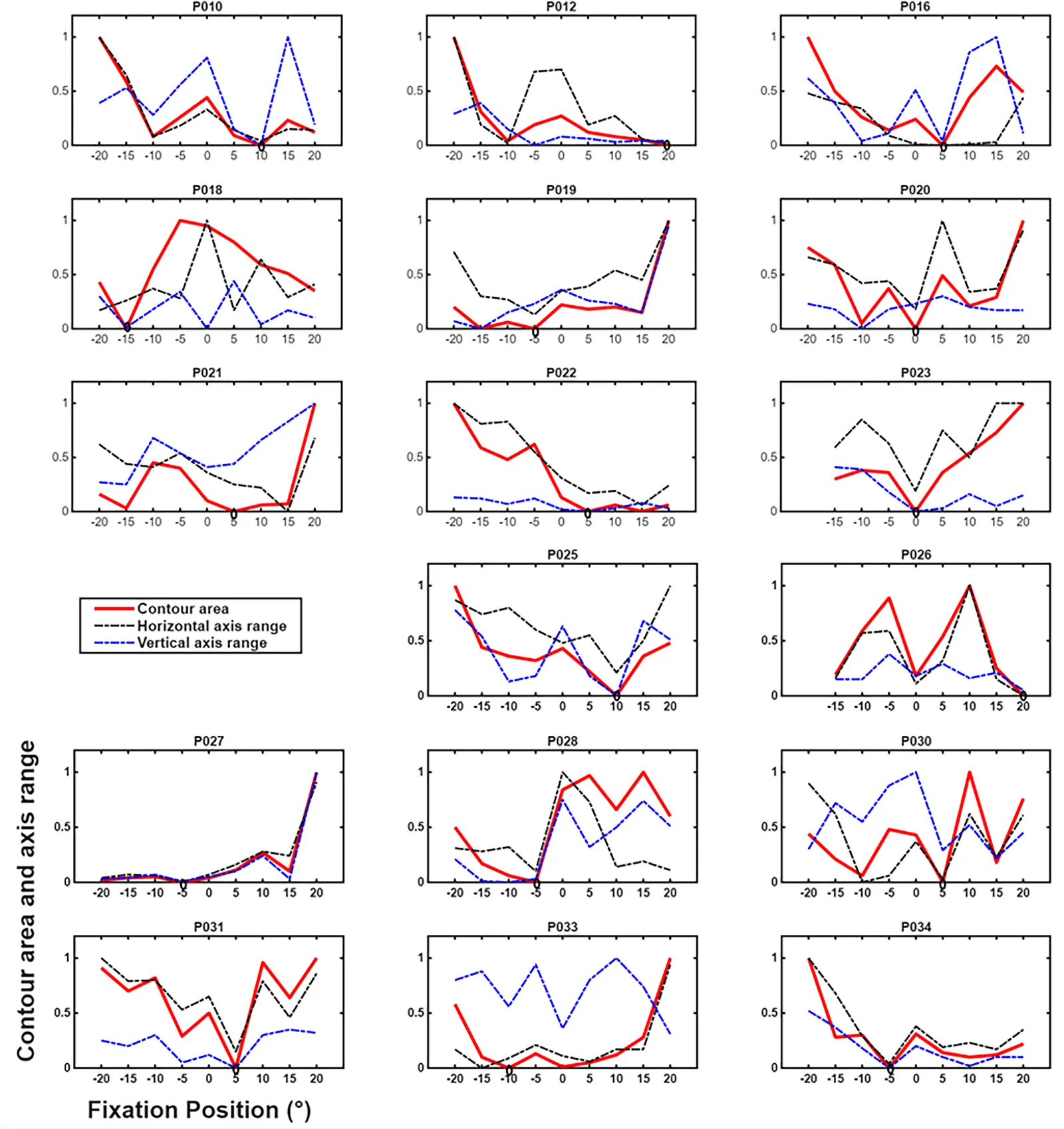
Isocontour area alongside the derived horizontal and vertical range at each gaze angle. Black ovals indicate the point of lowest contour area value where the eye positions spread less.

**Table 2.** Fixation Performance Across Gaze Angles Using Actual Values

	Mean ± SD				
Participant	Accuracy (°)	Contour Shape	Contour Area (deg2)	Horizontal Axis Range (°)	Vertical Axis Range (°)
P010	1.70 ± 0.75	0.49 ± 0.22	4.47 ± 3.09	3.65 ± 2.62	1.81 ± 0.47
P012	2.18 ± 1.70	0.46 ± 0.21	15.68 ± 14.44	6.05 ± 3.59	3.75 ± 1.34
P016	3.99 ± 1.63	0.38 ± 0.27	11.34 ± 6.63	3.68 ± 2.29	5.96 ± 3.99
P018	1.68 ± 1.46	0.50 ± 0.22	7.32 ± 1.86	4.14 ± 1.78	2.67 ± 1.04
P019	1.77 ± 0.87	0.72 ± 0.13	3.80 ± 1.98	2.41 ± 0.54	2.01 ± 0.57
P020	0.79 ± 0.56	0.56 ± 0.15	3.34 ± 0.90	2.71 ± 0.73	1.74 ± 0.22
P021	8.07 ± 2.74	0.45 ± 0.17	25.06 ± 16.25	5.52 ± 1.78	6.96 ± 2.08
P022	1.93 ± 1.38	0.34 ± 0.18	12.78 ± 9.26	7.55 ± 4.47	2.38 ± 0.61
P023	3.95 ± 1.47	0.33 ± 0.15	5.17 ± 2.03	4.37 ± 1.21	2.08 ± 0.70
P025	2.25 ± 1.13	0.56 ± 0.14	11.06 ± 3.58	4.42 ± 0.76	3.68 ± 0.89
P026	2.89 ± 1.95	0.68 ± 0.22	4.14 ± 2.45	2.72 ± 1.55	1.95 ± 0.47
P027	1.84 ± 2.59	0.65 ± 0.24	4.49 ± 6.28	2.68 ± 2.28	2.44 ± 2.57
P028	1.75 ± 0.53	0.50 ± 0.19	4.52 ± 1.76	2.78 ± 1.21	2.74 ± 1.18
P030	6.92 ± 4.10	0.36 ± 0.23	21.53 ± 9.40	6.01 ± 3.14	7.63 ± 2.65
P031	1.59 ± 0.58	0.49 ± 0.12	11.54 ± 4.20	5.36 ± 1.39	2.87 ± 0.67
P033	2.69 ± 1.87	0.25 ± 0.12	17.88 ± 9.03	3.96 ± 2.76	8.84 ± 2.42
P034	1.56 ± 0.90	0.50 ± 0.20	6.79 ± 5.55	4.14 ± 2.29	2.64 ± 1.32

The horizontal and vertical axis ranges at each gaze position were derived from contour area, contour shape, and the major axis orientation.

Individual plots of normalized values show that, across horizontal gaze positions, the points of best fixation accuracy, best precision, and most symmetric isocontour (i.e., highest shape factor, indicating less disparity in the horizontal and vertical waveform components) do not always correspond. Exceptions were participants P010, P018, P019, P022, P025, P027, P028, P030, P031, and P033, where the best points for these variables were identified on the same side, either left and right. The isocontour shape factor (contour shape) appeared to vary more across eccentric gaze positions than other measured parameters. Although higher isocontour symmetry only highlights almost equal major and minor isocontour axis ranges, as seen in the plots, this finding may not always signify better fixation performance in terms of accuracy and precision.

### Horizontal and Vertical Axis Ranges of Data Spread

Using normalized values, results for the horizontal and vertical axis range of data spread are shown as a function of gaze angle in Figure 5. The individual graphs indicate that about half of the participants (eight out of 17) had the least point of data spread at the vertical axis range, corresponding to the point of lowest contour area value. These were participants P010, P018, P022, P023, P025, P028, P031, and P034.

Findings from the linear mixed-effects model indicate that there was no significant linear effect of gaze angle (β = −0.038, *P* = 0.052), but a significant quadratic effect of gaze angle on the axis of fixation instability, as the horizontal and vertical axis range of the 68% isocontour varied with gaze angle (β = 0.005, *P* < 0.001).

Although there was a significant difference between the mean horizontal (4.25° ± 2.58°) and vertical (3.65° ± 2.72°) axis range (*U* = 13928, *P* = 0.000865), using the linear mixed model, no significant main effect of axis was found (β = 0.016, *P* = 0.964). “Axis” was coded as a binary variable, such that β estimates represent the vertical axis relative to the horizontal axis. Significant interaction effects indicated that the relationship between gaze angle and fixation instability differed between the horizontal and vertical axes (angle × axis: β = 0.039, *P* = 0.035; angle^2^ × axis: β = −0.004, *P* = 0.026).

To summarize the variation in the horizontal and vertical axis range of IN waveform across participants, the relationships between the axis range of vertical and horizontal eye positions, as well as other computed parameters are presented in Table 2. As expected, the results indicate that, for those in whom the horizontal and vertical axis ranges were almost equal, the isocontours were more circular (shape factor closer to 1, such as P019 and P026). In others for whom isocontours were less circular (shape factor closer to 0, such as P023 and P033), the horizontal and vertical axis ranges differed, indicating a difference in the vertical spread of data from the horizontal. Other values of accuracy and precision show variability among participants and within participants, at different gaze angles.

Furthermore, the gaze angles suggesting null zones are presented in Table 3. With reference to the wave form dynamics, the null zone was taken as the gaze angle with the lowest amplitude, lowest frequency, and lowest intensity. These values varied with each individual. Points of highest precision (lowest contour area value) can also be found in this table. In four participants (P010, P020, P028, and P034), the gaze angle of highest precision corresponded to the gaze angle with least amplitude, least frequency, or least intensity. A Bland–Altman plot (Fig. 6) further showed a mean difference of 0.29° (95% confidence interval [CI], −7.55 to 8.14), a lower boundary of −29.61° (95% CI, −43.19 to −16.02), and an upper boundary of 30.19° (95% CI, 16.61–43.78). Although the mean difference or bias between both measures of intensity and precision is small, the confidence limits of agreement are large.

**Table 3.** Gaze Positions Indicating Least Measured Parameters

Participant	Gaze Angle of Least Amplitude (°)	Gaze Angle of Least Frequency (°)	Gaze Angle of Least Intensity (°)	Gaze Angle of Least Contour Area (Highest Precision) (°)
P010	20	**10**	**10**	**10**
P012	15	10	15	20
P016	0	−20	0	5
P018	20	5	20	−15
P019	−10	5	−10	−5
P020	5	**0**	**0**	**0**
P021	−20	−15	0	5
P022	15	20	20	5
P023	10	20	10	0
P025	5	15	5	10
P026	15	−20	−20	20
P027	5	5	5	−5
P028	0	−**5**	0	−**5**
P030	−10	−5	−5	5
P031	−5	−20	−5	5
P033	5	0	0	−10
P034	−**5**	0	0	−**5**

Values with a negative sign indicate gaze angles to the left gaze. Corresponding values of amplitude, frequency, intensity, and contour area are highlighted in bold.

**Figure 6.**
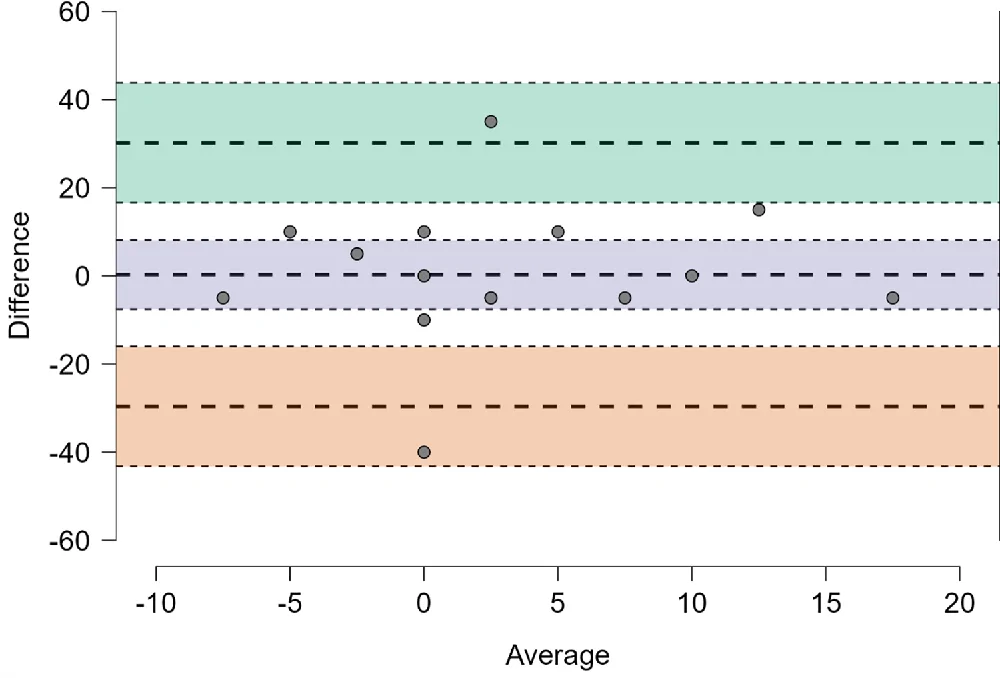
Bland–Altman plot evaluating gaze angle of least waveform intensity and highest precision.

### Fixation Performance and Clinical VA

As a follow up to data from Table 2, the mean accuracy and precision values obtained within each participant were correlated with their clinical VA. The graphs in Figure 7 illustrate these findings. Results indicate a positive correlation between mean accuracy and VA (*r* = 0.659; *P* = 0.004; 95% CI, 0.262–0.866), as well as mean precision and VA (*r* = 0.677; *P* = 0.003; 95% CI, 0.291–0.873). In other words, a decrease in VA is associated with less accurate fixation and less precise fixational eye position (i.e., mean contour area) (Figure 7a). Furthermore, although the VA of participants with idiopathic IN appeared to be better than among those without idiopathic IN, Figure 7b shows that those with idiopathic IN, who made up about half of the participants (47%), did not have a significantly better VA and precision than others (*t* = 1.83, *P* = 0.11).

**Figure 7.**
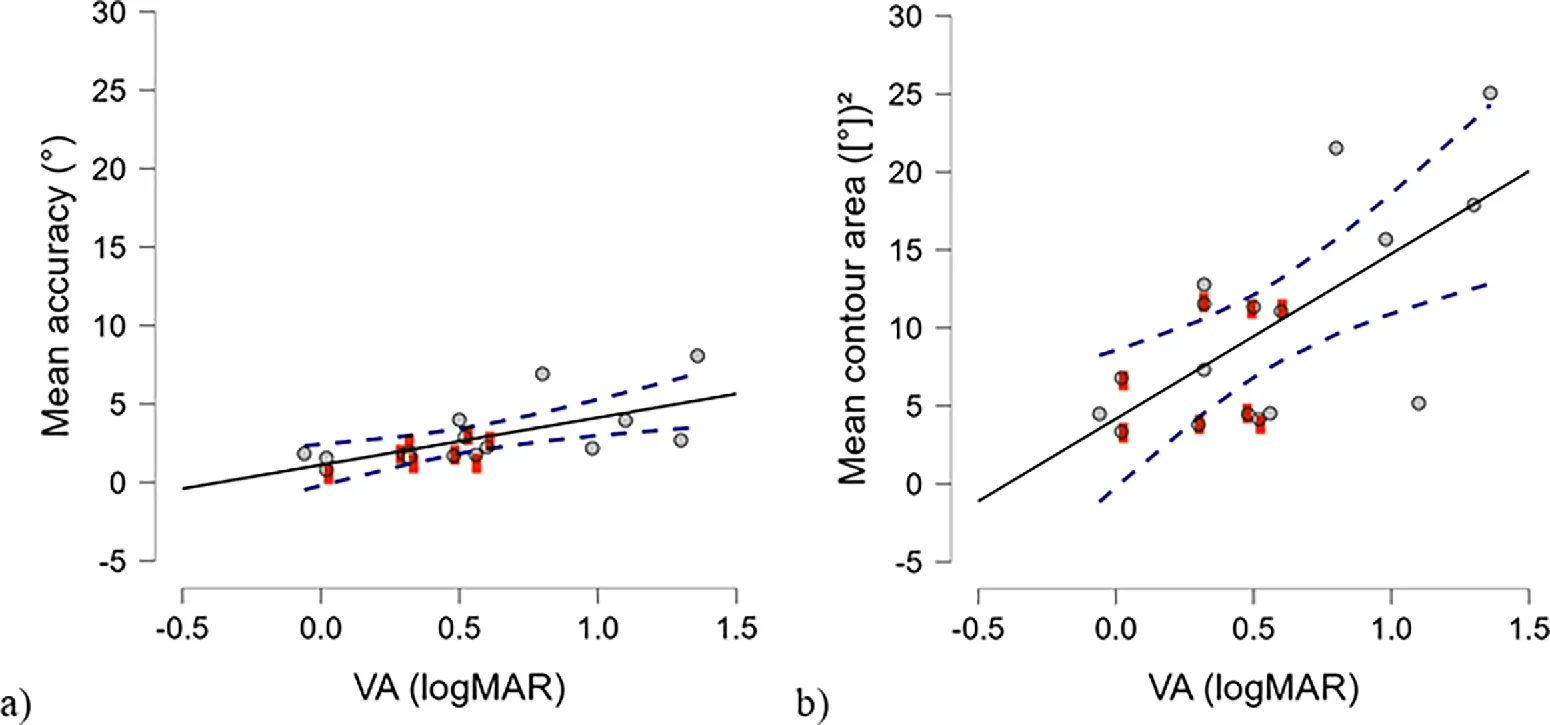
The relationship between fixation performance and clinical VA. (**a**) Data obtained from the mean accuracy across gaze positions, in each participant. (**b**) The mean contour area (precision) across all gaze positions for each participant. *Blue dotted lines* indicate the 95% CI regions. *Red* *spots* represent values from the eight idiopaths. A higher logMAR value indicates poorer VA.

## Discussion

This study aimed to quantify fixation performance in participants with IN, at eccentric gaze positions, using the bPDF approach. Unlike previous studies,^26^^,^^37^ here we describe fixation performance two-dimensionally in terms of the stability of eye movements. We believe this provides a more complete quantification of the distribution of eye positions produced by the IN waveform, as it evaluates the horizontal and vertical IN waveforms together, rather than separately. Because the isocontour area is comprised of both horizontal and vertical eye position spread, the contribution of vertical eye position to fixational eye stability for people with IN is included.

Our findings indicate high variability in fixation performance, both between and within participants with IN, when fixating at different gaze angles. This variability was evident in the measured accuracy and precision of fixation. Many people with IN are known to prefer using their null zone, but why they do so is unclear.^16^^,^^23^ If we were to attempt to identify an individual's objective null zone using the accuracy and precision data, such a conclusion would be difficult to reach, because in almost all participants (except P025) the most accurate (shortest distance of isocontour to fixation point) gaze angle was not always the most precise (smallest isocontour area). This may be because, at some positions of gaze, the IN eye movement beat direction may flip. Considering the resulting isocontour, fixation may remain accurate; however, data points may occupy a larger area and therefore become less precise.

Furthermore, as illustrated in the individual plots of Figure 5, the points where contour area are lowest do not always give an equivalent reduction in both horizontal and vertical components. In most cases, measured values for both components do not correspond, and this raises the question as to how much precision of eye movement is determined by the minimum overall movement (i.e., contour area), the minimum horizontal movement, or the minimum vertical movement. Considering the traditional method of determining null zone using intensity and the findings of eye movement precision, a small mean bias of 0.29° was found using the Bland–Altman plot. Although this resulting mean difference between both methods was small, the wide limits of agreement indicate substantial inter-individual variability, which may partly reflect the restricted gaze range tested. In this study, the sampled gaze range was limited to ±20° in 5° steps, meaning that, for some participants, the true optimum gaze position (based on least waveform intensity or greater fixation precision) may have fallen outside the tested range, potentially exaggerating the disagreement between methods. Nonetheless, these findings indicate that measures of traditional waveform metrics and fixation precision are related but may not necessarily be an equivalent indication of null zone behavior. These findings highlight the fact that traditional methods that evaluate only horizontal eye position to characterize the null zone may miss important information about how the vertical components may have contributed to the null zone findings.

Attempts to understand the contributions of the vertical component are complicated by the fact that findings from this experiment do not provide a clear pattern of how the vertical components contribute to best accuracy and precision. In some cases, the horizontal and vertical components change together. This finding has been observed clinically and is indeed similar to that found by Bedell and colleagues,^26^ in which the variability of eye position in the horizontal and vertical components for all their participants was positively related. Because our present study evaluated eye position variability two dimensionally and at varying gaze angles, our results are not directly comparable to theirs.

Furthermore, in most of the participants from the present study, the vertical range was largely unaffected by the degree of horizontal eye movement, whereas in a couple of participants there was a dramatic effect. As shown from the linear mixed model results, findings indicate that the horizontal and vertical axis range significantly differed with respect to gaze angle. As gaze angle interacts significantly with axis, the horizontal axis range was shown to be affected more strongly than the vertical axis range at the more extreme gaze angles. This suggests that there was more horizontal that vertical spread of eye movement recordings at the more extreme gaze angle. In addition, changes in the axis range values at each gaze angle generally suggest a heterogeneity in the changes between the horizontal and vertical components. These findings pose the interesting possibility that the AHP in some individuals with IN might reflect the need to compensate for the vertical as well as the horizontal component of their oscillatory eye movements. However, determining this will require further investigation into the extent to which AHPs relate to the vertical waveform component. There is evidence that an AHP (chin elevation/depression) can be used to achieve null zones in IN.^54^ Therefore, it can be argued that achieving null zones may not exclusively be a “horizontal-only” phenomenon.^3^^,^^20^^,^^55^ However, care should be taken when interpreting the data, as findings point to a variability in the relationship between the horizontal and vertical components warranting further investigation. Because video-based eye tracking cannot measure any torsional component to the oscillation, a complete characterization of the null zone remains to be determined. Although exploratory, the present findings contribute to current knowledge about the nature of eye movements in IN at different gaze positions, suggesting that differences in waveform components alone may not explain fixation preference at the null zone.

The impact of both aspects of fixation performance (as computed using the bPDF method) on clinically measured VA is reflected in their significant positive correlation. The span of eye movement in the derived bPDF isocontour may reflect the amplitude of a given waveform, albeit in two dimensions, and it is this degree of eye movement excursion that is judged clinically when doing a clinically observed objective null zone assessment. The fact that, across participants, VA correlates significantly with fixation accuracy and precision is in line with previous studies that the visual threshold gets poorer when nystagmus intensity increases.^16^^,^^23^ This is because eye velocity is known to reduce during foveation in people with IN.^19^^,^^26^ However, it is worth noting that data reported here may not necessarily mean that, for any given individual with IN, treatments that reduce nystagmus intensity would improve VA.^16^ To further probe the effects of waveform changes and the null zone on VA, it will be necessary to examine whether VA changes with gaze angles and its relationship with fixation performance using horizontal and vertical eye movements. This will be addressed in a follow-up study.

The bPDF contour area (precision) represents the variability of eye movement during fixation and is likely to reflect the underlying VA. The findings of fixation performance, specifically its precision, and its relationship with VA are useful for clinicians and researchers. The significant association of greater inaccuracy and imprecision with poorer VA may provide the basis for predicting future VA in a child, given that there is no evidence that nystagmus characteristics change throughout life beyond the ages of 3 to 5 years. For most children, the IN waveform stabilizes from about age 3 years to just before age 5 years,^56^^,^^57^ when they start learning to read.^58^ This predicted level of vision might be able to be used to provide early schooling support that is appropriate for their level of vision and may be beneficial throughout their learning process. Of course, future studies will be necessary to demonstrate the predictive power of these measures.

This study has highlighted the importance of including the vertical component of IN eye movements during analysis of attempted fixation to provide a better explanation of the basis for null zone preference. Future research would benefit from including a two-dimensional approach to the analysis of IN eye movements to provide a more holistic picture of what the eyes are doing at any given time, especially with respect to gaze angles.

## References

[1] Gottlob I, Zubcov A, Catalano RA, et al. Signs distinguishing spasmus nutans (with and without central nervous system lesions) from infantile nystagmus. *Ophthalmology*. 1990; 97(9): 1166–1175. 10.1016/s0161-6420(90)32440-5 2234849

[2] Abadi RV, Dickinson CM. Waveform characteristics in congenital nystagmus. *Doc Ophthalmol*. 1986; 64: 153–167. 10.1007/BF00159990 3608756

[3] Abadi RV, Bjerre A. Motor and sensory characteristics of infantile nystagmus. *Br J Ophthalmol*. 2002; 86(10): 1152–1160. 10.1136/bjo.86.10.1152 PMC1771304 12234898

[4] Sarvananthan N, Surendran M, Roberts EO, et al. The prevalence of nystagmus: the Leicestershire nystagmus survey. *Invest Ophthalmol Vis Sci*. 2009; 50(11): 5201–5206. 10.1167/iovs.09-3486 19458336

[5] Gu S, Hu Y, Zhao Y, et al. A retrospective study on the eye-related quality of life, functional vision, and their determinants among children following congenital and developmental cataracts surgery and its impact on their families using the PedEyeQ. *Front Public Health*. 2022; 10: 788384. 10.3389/fpubh.2022.788384 PMC8968105 35372237

[6] Pilling RF, Thompson JR, Gottlob I. Social and visual function in nystagmus. *Br J Ophthalmol*. 2005; 89(10): 1278–1281. 10.1136/bjo.2005.070045 PMC1772873 16170116

[7] Kraft SP, Irving EL. A case of different null zones for distance and near fixation. *Am Orthopt J*. 2004; 54(1): 102–111. 10.3368/aoj.54.1.102 21149093

[8] Hertle RW, Dell'Osso LF. Clinical and ocular motor analysis of congenital nystagmus in infancy. *J AAPOS*. 1999; 3(2): 70–79. 10.1016/s1091-8531(99)70073-x 10221798

[9] Thurtell MJ, Leigh RJ. Treatment of nystagmus. *Curr Treat Options Neurol*. 2012; 14(1): 60–72. 10.1007/s11940-011-0154-5 22072056

[10] Chang MY, Binenbaum G, Heidary G, et al. Surgical treatments to improve visual acuity in infantile nystagmus syndrome: a report by the American Academy of Ophthalmology. *Ophthalmology*. 2023; 130(3): 331–344. 10.1016/j.ophtha.2022.10.006 PMC9979093 36435636

[11] Serra A, Dell'Osso LF, Jacobs JB, Burnstine RA. Combined gaze-angle and vergence variation in infantile nystagmus: two therapies that improve the high-visual-acuity field and methods to measure it. *Invest Ophthalmol Vis Sci*. 2006; 47(6): 2451–2460. 10.1167/iovs.05-1320 16723456

[12] Dell'Osso LF . Development of new treatments for congenital nystagmus. *Ann N Y Acad Sci*. 2002; 956: 361–379. 10.1111/j.1749-6632.2002.tb02834.x 11960819

[13] Roberts TL, Kester KN, Hertle RW. Reliability and validity of gaze-dependent functional vision space: a novel metric quantifying visual function in infantile nystagmus syndrome. *Invest Ophthalmol Vis Sci*. 2018; 59(5): 1760–1768. 10.1167/iovs.17-23229 PMC5886028 29610862

[14] Fadzil NM, Mohammed Z, Shahimin MM, Saliman NH. Reading performance and compensatory head posture in infantile nystagmus after null zone training. *Int J Environ Res Public Health*. 2019; 16(23): 1–7. 10.3390/ijerph16234728 PMC6926938 31783492

[15] Fitzmaurice K, Lesley T. Visual acuity is not the bottom line: some techniques of visual rehabilitation. *Aust Orthopt J*. 1987; 24: 23–25.

[16] Dunn MJ, Wiggins D, Woodhouse JM, Margrain TH, Harris CM, Erichsen JT. The effect of gaze angle on visual acuity in infantile nystagmus. *Invest Ophthalmol Vis Sci*. 2017; 58(1): 642–650. 10.1167/iovs.16-20370 28129427

[17] da Costa ACRV, Lopes MCB, Nakanami CR. Influence of head posture on the visual acuity of children with nystagmus. *Arq Bras Oftalmol*. 2014; 77(1): 8–11. 10.5935/0004-2749.20140004 25076365

[18] Dell'Osso LF, Van Der Steen J, Steinman RM, Collewijn H. Foveation dynamics in congenital nystagmus I: Fixation. *Doc Ophthalmol*. 1992; 79: 1–23. 10.1007/BF00160130 1568420

[19] Dell'Osso LF, Daroff RB. Congenital nystagmus waveform and foveation strategy. *Doc Ophthalmol*. 1975; 39(1): 155–182. 10.1007/BF00578761 1201697

[20] Abadi RV, Whittle J. The nature of head postures in congenital nystagmus. *Arch Ophthalmol*. 1991; 109(2): 216–220. 10.1001/archopht.1991.01080020062044 1993030

[21] Dell'Osso L, Gauthier G, Liberman G, Stark L. Eye movement recordings as a diagnostic tool in a case of congenital nystagmus. *Am J Optom Arch Am Acad Optom*. 1972; 49(1): 3–13. 10.1097/00006324-197201000-00002 4500610

[22] Jones PH, Harris CM, Woodhouse JM, Margrain TH, Ennis FA, Erichsen JT. Stress and visual function in infantile nystagmus syndrome. *Invest Ophthalmol Vis Sci*. 2013; 54(13): 7943–7951. 10.1167/iovs.13-12560 24222308

[23] Wiggins D, Woodhouse JM, Margrain TH, Harris CM, Erichsen JT. Infantile nystagmus adapts to visual demand. *Invest Ophthalmol Vis Sci*. 2007; 48(5): 2089–2094. 10.1167/iovs.06-1108 17460265

[24] Collewijn H, Apkarian P, Spekreijse H. The oculomotor behaviour of human albinos. *Brain*. 1985; 108(1): 1–28. 10.1093/brain/108.1.1 3978393

[25] Thomas MG, Gottlob I, McLean RJ, Maconachie G, Kumar A, Proudlock FA. Reading strategies in infantile nystagmus syndrome. *Invest Ophthalmol Vis Sci*. 2011; 52(11): 8156–8165. 10.1167/iovs.10-6645 21862655

[26] Bedell HE, White JM, Abplanalp PL. Variability of foveations in congenital nystagmus. *Clin Vision Sci*. 1989; 4(3): 247–252.

[27] Dell'Osso LF, Van Der Steen J, Steinman RM, Collewijn H. Foveation dynamics in congenital nystagmus. II: Smooth pursuit. *Doc Ophthalmol*. 1992; 79: 25–49. 10.1007/BF00160131 1568421

[28] Dell'Osso LF, Van Der Steen J, Steinman RM, Collewijn H. Foveation dynamics in congenital nystagmus IV: vergence. *Doc Ophthalmol*. 2020; 140(3): 221–232. 10.1007/s10633-019-09738-y 31776760

[29] Abadi RV, Pascal E, Whittle J, Worfolk R. Retinal fixation behavior in human albinos. *Optom Vision Sci*. 1989; 66(5): 276–280. 10.1097/00006324-198905000-00004 2748105

[30] Dell'Osso LF, Flynn JT, Daroff RB. Hereditary congenital nystagmus. An intrafamilial study. *Arch Dis Child*. 1974; 92: 366–374. 10.1001/archopht.1974.01010010378002 4429465

[31] Felius J, Fu VLN, Birch EE, Hertle RW, Jost RM, Subramanian V. Quantifying nystagmus in infants and young children: relation between foveation and visual acuity deficit. *Invest Ophthalmol Vis Sci*. 2011; 52(12): 8724–8731. 10.1167/iovs.11-7760 22003105

[32] Gresty M, Page N, Barratt H. The differential diagnosis of congenital nystagmus. *J Neurol Neurosurg Psychiatry*. 1984; 47(9): 936–942. 10.1136/jnnp.47.9.936 PMC1027994 6481387

[33] Ukwade MT, Bedell HE. Variation of congenital nystagmus with viewing distance. *Optom Vision Sci*. 1992; 69(12): 976–985. 10.1097/00006324-199212000-00012 1300523

[34] Dell'Osso LF, Flynn JT. Congenital nystagmus surgery. A quantitative evaluation of the effects. *Arch Ophthalmol*. 1979; 97: 462–469. 10.1001/archopht.1979.01020010212004 420633

[35] Castet E, Crossland M. Quantifying eye stability during a fixation task: a review of definitions and methods. *Seeing Perceiving*. 2012; 25(5): 449–469. 10.1163/187847611X620955 22370759

[36] Dell'Osso LF, Jacobs JB. An expanded nystagmus acuity function: intra-and intersubject prediction of best-corrected visual acuity. *Doc Ophthalmol*. 2002; 104: 249–276. 10.1023/a:1015299930849 12076015

[37] Dell'Osso LF . Fixation characteristics in hereditary congenital nystagmus. *Am J Optom Arch Am Acad Optom*. 1973; 50(2): 85–90. 4511863

[38] Dai B, Cham KM, Abel LA. Velocity discrimination in infantile nystagmus syndrome. *Invest Ophthalmol Vis Sci*. 2021; 62(10): 1–9. 10.1167/iovs.62.10.35 PMC8411855 34459850

[39] Dunn MJ, Harris CM, Ennis FA, et al. An automated segmentation approach to calibrating infantile nystagmus waveforms. *Behav Res Methods*. 2019; 51(5): 2074–2084. 10.3758/s13428-018-1178-5 PMC6797654 30875024

[40] Thaler L, Schütz AC, Goodale MA, Gegenfurtner KR. What is the best fixation target? The effect of target shape on stability of fixational eye movements. *Vision Res*. 2013; 76: 31–42. 10.1016/j.visres.2012.10.012 23099046

[41] Brainard DH. The Psychophysics Toolbox. *Spat Vis*. 1997; 10(4): 433–436. 9176952

[42] Pelli DG. The VideoToolbox software for visual psychophysics: transforming numbers into movies. *Spat Vis*. 1997; 10(4): 437–442. 9176953

[43] Kleiner M, Brainard D, Pelli D. What's new in Psychtoolbox-3?. *Perception*. 2007; 36(suppl): 14.

[44] SR Research. EyeLink Eye Tracker Learning Resources. Available at: https://www.sr-research.com/support-options/learning-resources/. Accessed September 2, 2026.

[45] Davies JR, Freeman TCA. Simultaneous adaptation to non-collinear retinal motion and smooth pursuit eye movement. *Vision Res*. 2011; 51(14): 1637–1647. 10.1016/j.visres.2011.05.004 21605588

[46] Mack DJ, Belfanti S, Schwarz U. The effect of sampling rate and lowpass filters on saccades – a modeling approach. *Behav Res Methods*. 2017; 49(6): 2146–2162. 10.3758/s13428-016-0848-4 28130727

[47] Butterworth S. On the theory of filter amplifiers. *Exp Wirel & Wirel Eng*. 1930; 7: 536–541.

[48] Cherici C, Kuang X, Poletti M, Rucci M. Precision of sustained fixation in trained and untrained observers. *J Vis*. 2012; 12(6): 1–16. 10.1167/12.6.31 PMC3489479 22728680

[49] McIlreavy L, Freeman TCA, Erichsen JT. Two-dimensional analysis of smooth pursuit eye movements reveals quantitative deficits in precision and accuracy. *Transl Vis Sci Technol*. 2019; 8(5): 1–17. 10.1167/tvst.8.5.7 PMC6753966 31588372

[50] McIlreavy L, Freeman TCA, Erichsen JT. Two-dimensional analysis of horizontal and vertical pursuit in infantile nystagmus reveals quantitative deficits in accuracy and precision. *Invest Ophthalmol Vis Sci*. 2020; 61(6): 1–12. 10.1167/iovs.61.6.15 PMC7415281 32526031

[51] Botev ZI, Grotowski JF, Kroese DP. Kernel density estimation via diffusion. *Ann Stat*. 2010; 38(5): 2916–2957.

[52] Botev ZI. Kernel density estimation - file exchange - MATLAB Central. Available at: https://uk.mathworks.com/matlabcentral/fileexchange/17204-kernel-density-estimation?s_tid=srchtitle. Accessed May 21, 2023.

[53] Crossland MD, Rubin GS. The use of an infrared eyetracker to measure fixation stability. *Optom Vision Sci*. 2002; 79(11): 735–739. 10.1097/00006324-200211000-00011 12462542

[54] Al-Dabet S, Turaev S, Zaki N. Ocular-induced abnormal head postures: a systematic review and analysis. *Surv Ophthalmol*. 2025; 70(4): 771–816. 10.1016/j.survophthal.2025.01.016 39933628

[55] Maybodi M. Infantile-onset nystagmus. *Curr Opin Ophthalmol*. 2003; 14(5): 276–285. 10.1097/00055735-200310000-00008 14502055

[56] Hussain N. Diagnosis, assessment and management of nystagmus in childhood. *Paediatr Child Health*. 2016; 26(1): 31–36.

[57] Hertle RW. Nystagmus in infancy and childhood. *Semin Ophthalmol*. 2008; 23(5-6): 307–317. 10.1080/08820530802505955 19085433

[58] Simons HD. An analysis of the role of vision anomalies in reading interference. *Optom Vision Sci*. 1993; 70(5): 369–373. 10.1097/00006324-199305000-00005 8515964

